# Cottage Hospital Expenditure

**Published:** 1898-10-22

**Authors:** 


### COTTAGE HOSPITAL EXPENDITURE.

It is a good sign when the annual meeting of a cottage hospital is largely attended, and when the governors present show by their remarks an intelligent and practical interest in the administration and welfare of their institution. The Devizes Cottage Hospital is to be congratulated upon such a meeting, and upon the discussion which took place in the course of it. The Rev. W. Gardiner offered some criticisms upon the expenditure, and pointed out that if proper care were exercised experience proved that 8s. per week per patient was an adequate sum to expend, and that a saving of some £65 per annum on the maintenance of patients alone might be effected if the system were altered. Mr. Gardiner has evidently made himself practically acquainted with the facts, for he made two good points. First of all, he insisted upon the importance of adopting the Uniform System of Accounts, holding it was most desirable that the reports of various cottage hospitals should be cast in the same form. He further insisted, quite rightly, that the whole of the money received in dividends should be credited in the hospital accounts, and that returned income-tax should be entered separately. We are a little surprised to find that Mr. Owen, the secretary, stated in reply that he was waiting for some form of accounts to be generally adopted by hospitals. Surely Mr. Owen must have heard of the Unform System, and if he has not he can ascertain all about it by sending to the Scientific Press, 28 and 29, Southampton Street, Strand, London, W.C. As our readers know, the Uniform System has been adapted to cottage hospital book-keeping, and a set of books may be obtained at a small outlay which would meet the whole of the points so wisely insisted upon by Mr. Gardiner. As Mr. Owen admits the advantage of making a change in order to keep the books upon a common form used by other hospitals, we have no doubt he will speedily introduce the Uniform System at Devizes Cottage Hospital. We would also strongly urge Mr. Owen to amend the loose system at present adopted in regard to the return of income-tax. It has occurred over and over again in the history of hospitals and kindred institutions that, when the dividends are treated as paid in full without deduction of income-tax, and where no separate item is given regarding the return of such tax as and when received, there the time is sure to come when the return of income-tax is not claimed, and so the charity suffers pecuniary loss. We hope Mr. Gardiner will, therefore, persist in his point, and see that his suggestion takes practical effect without delay. Having regard to the fact that the out-patients are three times as numerous as the in-patients, owing to a dispensary being attached to the cottage hospital, we are strongly of opinion that the expenditure upon the dispensary should be kept entirely distinct from that of the hospital, and that two separate accounts should be rendered. This has been done at other cottage hospitals where dispensaries are attached, and until some such system is adopted it is impossible to ascertain accurately and satisfactorily how much money is expended upon in-patients and how much it costs to maintain the dispensary branch of the institution. Whilst congratulating the governors of Devizes Cottage Hospital upon the intelligent interest they have shown in their excellent institution, we venture to hope that before the next annual meeting the changes advocated by Mr. Gardiner, which we have here supported, will be introduced.

